# Expression of immune related genes and possible regulatory mechanisms in different stages of non-alcoholic fatty liver disease

**DOI:** 10.3389/fimmu.2024.1364442

**Published:** 2024-03-08

**Authors:** Risheng He, Canghai Guan, Xudong Zhao, Liang Yu, Yunfu Cui

**Affiliations:** Department of Pancreatobiliary Surgery, The Second Affiliated Hospital of Harbin Medical University, Harbin, Heilongjiang, China

**Keywords:** non-alcoholic fatty liver disease, weighted gene co-expression network analysis, immunity-related genes, immune infiltration, hub genes

## Abstract

**Background:**

Non-alcoholic fatty liver disease (NAFLD), which includes simple steatosis (SS) and non-alcoholic steatohepatitis (NASH), is a significant contributor to liver disease on a global scale. The change of immunity-related genes (IRGs) expression level leads to different immune infiltrations. However, the expression of IRGs and possible regulatory mechanisms involved in NAFLD remain unclear. The objective of our research is to investigate crucial genes linked to the development of NAFLD and the transition from SS to NASH.

**Methods:**

Dataset GSE89632, which includes healthy controls, SS patients, and NASH patients, was obtained using the GEO database. To examine the correlation between sets of genes and clinical characteristics, we employed weighted gene co-expression network analysis (WGCNA) and differential expression analysis. Hub genes were extracted using a network of protein-protein interactions (PPI) and three different machine learning algorithms. To validate the findings, another dataset that is publicly accessible and mice that were subjected to a high-fat diet (HFD) or MCD diet were utilized. Furthermore, the ESTIMATE algorithm and ssGSEA were employed to investigate the immune landscape in the normal versus SS group and SS versus NASH group, additionally, the relationship between immune infiltration and the expression of hub genes was also examined.

**Results:**

A total of 28 immune related key genes were selected. Most of these genes expressed reverse patterns in the initial and progressive stages of NAFLD. GO and KEGG analyses showed that they were focused on the cytokine related pathways and immune cell activation and chemotaxis. After screening by various algorithms, we obtained two hub genes, including JUN and CCL20. Validation of these findings was confirmed by analyzing gene expression patterns in both the validation dataset and the mouse model. Ultimately, two hub genes were discovered to have a significant correlation with the infiltration of immune cells.

**Conclusion:**

We proposed that there were dynamic changes in the expression levels of IRGs in different stages of NAFLD disease, which led to different immune landscapes in SS and NASH. The findings of our research could serve as a guide for the accurate management of various phases of NAFLD.

## 1 Introduction

Over the past few decades, there has been a notable rise in the occurrence of metabolic disorders, such as obesity. It is crucial to note that obesity not only has its own associated comorbidities but also adversely affects overall health and increases susceptibility to other conditions (1, 2). As a result, people who are obese have an increased likelihood of experiencing comorbidities like insulin resistance, type 2 diabetes, high blood pressure, abnormal lipid levels, heart disease, and fatty liver disease. Non-alcoholic fatty liver disease (NAFLD) is acknowledged as the liver-related aspect of the metabolic syndrome (3). NAFLD is a widespread chronic liver disease that includes different conditions like simple steatosis (SS) and non-alcoholic steatohepatitis (NASH), which is characterized by inflammation (4). Projections indicate a significant rise in the prevalence of NAFLD, with the Chinese population estimated to exceed 300 million cases by 2030, over 100 million cases in the United States, and 15-20 million cases in major European countries (5). Hence, the escalating global incidence of NAFLD warrants considerable clinical scrutiny. Notably, the clinical presentation of NAFLD is inconspicuous during the stage of SS, only becoming apparent in the subsequent stage of NASH (6). As the disease advances, the liver undergoes significant pathological alterations, characterized by lobular inflammation and hepatocyte ballooning, accompanied by fibrosis in some cases (7). Failure to effectively manage the disease may lead to its progression to cirrhosis and, ultimately, liver cancer (8). Despite extensive research on this disease, its pathogenesis and the mechanisms underlying the progression from SS to NASH are still poorly understood.

In spite of the fact that it is primarily a metabolic disorder, inflammatory processes mediated by immune cells are involved in NAFLD, and inflammation is especially important at the stage of NASH, when it becomes integral to disease progression (9, 10). Increasing amounts of research validate the crucial involvement of the immune system in the different phases of NAFLD advancement, exhibiting alterations in immune cell infiltration levels and cytokine levels within the liver microenvironment throughout the progression of the disease (11, 12). In this study, we performed an extensive bioinformatics analysis comparing normal liver tissues with SS tissues, as well as SS tissues with NASH tissues. Based on comprehensive bioinformatics analyses, we have identified the immune status of the liver at different stages of the disease, as well as the hub genes and the mechanisms that regulate the immune microenvironment.

## 2 Materials and methods

### 2.1 Data acquisition and preliminary processing

**Figure 1:**
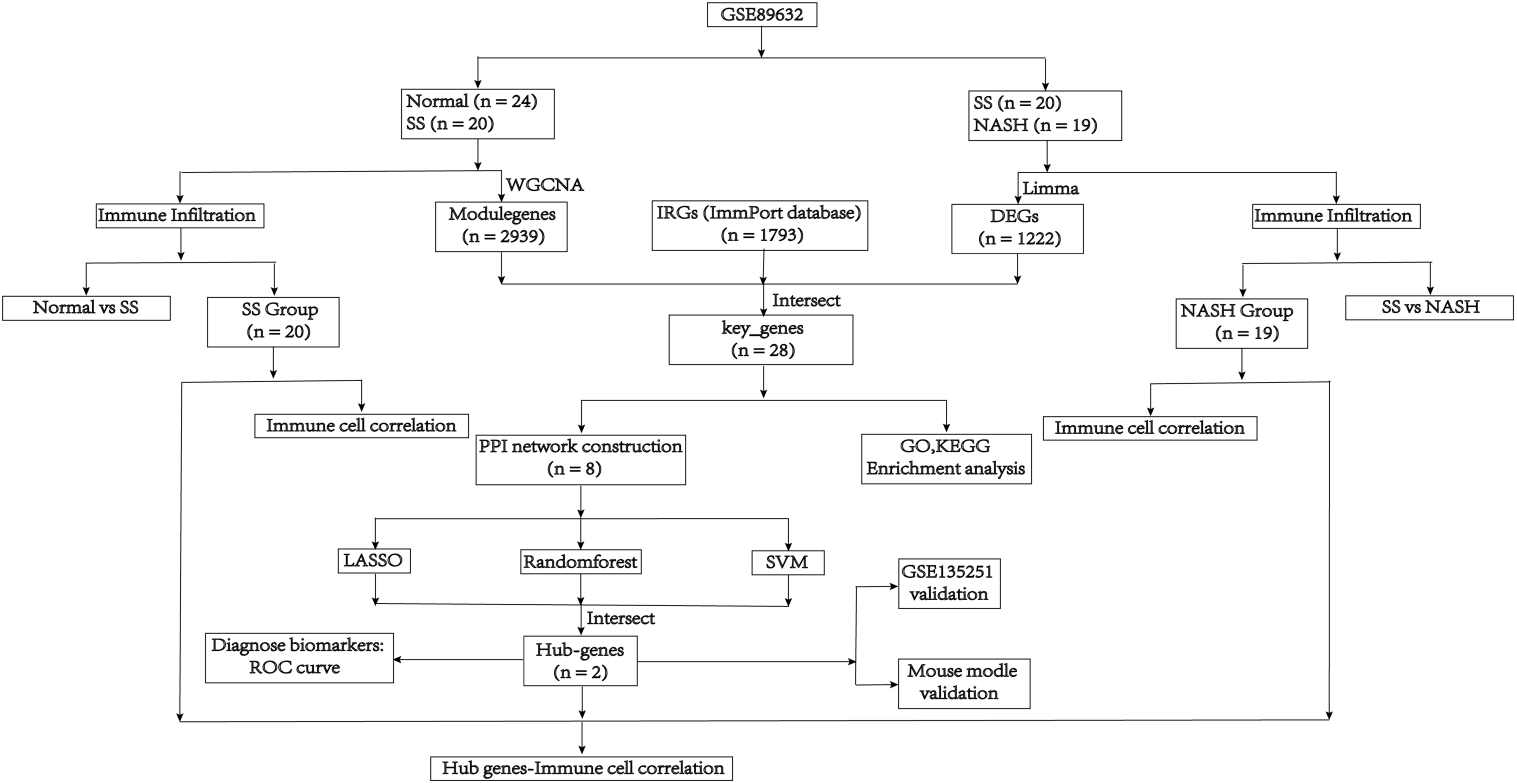
Flow diagram of the analysis process.

The mRNA sequencing dataset GSE89632 and GSE135251, which were obtained from the GEO database in NCBI (http://www.ncbi.nlm.nih.gov/geo/), included expression profiles that were generated using the platforms GPL14951 and GPL18573, respectively. These data sets contained normal samples, SS samples and NASH samples. And log2 was performed to process raw counts. To further investigate, we obtained immunity-related genes (IRGs) from ImmPort, a curated immune database used for the management and identification of genes related to immunity (13). **Figure 1** depicted the main search process of the article.

### 2.2 Weighted gene co-expression network analysis and module gene selection in normal vs. SS patients

The WGCNA method was used to construct gene coexpression networks and identify functional modules (14).The cut height was set to 120 and the goodSamplesGenes function was used to filter outlier samples, resulting in the construction of a scale-free coexpression network. The most highly expressed 10000 genes were selected for the following analysis. The pickSoftThreshold function determined an appropriate ‘soft’ threshold power (β) for calculating intergenic adjacency. Weight coexpression network used the blockwiseModules function. The function plotDendroAndColors visualized the clustering among samples. The labeledHeatmap function showed the correlation between the group and gene Modules. The function plotEigengeneNetworks displayed the association among different gene Modules. Finally, gene significance (GS) and module membership (MM) correlations were calculated, and the corresponding module gene information was extracted for further analysis.

### 2.3 Identification of differentially expressed genes between SS samples and NASH samples

Differentially expressed genes (DEGs) were selected from the SS vs. NASH group using the R limma package (15), based on the threshold criteria of |log2FC| > 0.25 and p-value < 0.05. Using the R software, the volcano plot for the DEGs and the expression heatmap for the top 15 up and top 15 down genes were created using the ‘ggplot2’ and ‘pheatmap’ packages, respectively. The draw_venn method in the tinyarray R package was used to display the key genes, which were obtained by intersecting the DEGs, modulegenes, and IRGs. The differential expression of these genes in normal vs. SS group and SS vs. NASH group was visualized using the ggplot2 and ComplexHeatmap packages. Furthermore, the corrplot library was employed to examine the associations among these genes.

### 2.4 Enrichment analysis

To perform enrichment analysis on important genes, we utilized the enrichGO and enrichKEGG functions from the R package clusterProfiler. The enrichGO function was employed for Gene Ontology (GO) analysis, while the enrichKEGG function was used for Kyoto Encyclopedia of Genes and Genomes (KEGG) pathway analysis (16). A p-value cutoff of 0.05 was applied. The R package ggplot2 and stringr were used for visualization.

### 2.5 Protein–protein interaction network construction

Using the online tool STRING (https://string-db.org/), a PPI network was built by considering all essential genes and applying a filter condition (combined score > 0.4). Afterwards, we obtained the interaction data and enhanced the PPI network using Cytoscape software to improve its visual representation (17, 18). The identification of significant gene clusters and the acquisition of cluster scores were achieved using Minimal Common Oncology Data Elements (MCODE), with filter criteria including a degree cut-off of 2, a node score cut-off of 0.2, a k-core of 2, and a maximum depth of 100. The cytoHubba plugins utilized the Maximal Clique Centrality (MCC) algorithm to determine the significance of genes within the primary gene cluster.

### 2.6 Screening of hub genes

Three machine learning algorithms, containing lasso regression (19), random forest (RF) (20) and SVM (21), were used to screen the hub genes from the most significant gene cluster screened by PPI. We used Lasso Cox regression to detect changes in regression coefficients of the important genes. The optimal parameter λ was determined using 10-fold cross-validation with the R package glmnet. Finally, we selected genes based on lambda.min. And we used the R package plotmo to visualize the coefficient contraction of LASSO Cox regression. The RF algorithm utilized the R package random Forest to assess the significance of genes in the most prominent gene cluster identified by PPI. The top five genes were selected. Support Vector Machines (SVM) is a supervised machine learning method used for regression or classification tasks, which necessitates a labeled training dataset SVM-RFE, a method in machine learning, trained a subset of characteristics from various groups to reduce the feature set and identify the most influential features. In the end, we identified the hub genes by intersecting the genes screened using the three algorithms.

### 2.7 Verification of hub genes expression and diagnostic efficacy

The expression level of the central genes was determined using the GSE89632 dataset, and the diagnostic value of these genes in distinguishing between the normal and SS groups, as well as the SS and NASH groups, was evaluated by constructing ROC curves using the pROC package. And the above results were verified with the GSE135251 dataset.

### 2.8 Immune infiltration analysis and correlation analysis

The ESTIMATE algorithm was utilized to deduce the immune cell infiltration by analyzing the transcriptome data and the immune microenvironment scores (22). The scores comprised of the immune score, the stromal score, and the estimate score. To better recognize the immune cell characteristics in tissue of normal vs. SS group and SS vs. NASH group, we compared the differences of immune cell subsets in the samples. The ssGSEA method was employed to compare the distinct composition of 28 immune cells. The means in the normal vs. SS group and SS vs. NASH group were compared using the t_test function from the R package rstatix. Subsequently, the results were visualized using the ggboxplot function from the R package ggpubr (23, 24). The correlation between the distribution of immune cells in SS and NASH patients was uncovered using the corrplot R package. Furthermore, we examined the relationship between the expression of hub genes and the infiltration of immune cells in SS and NASH patients.

### 2.9 Construction of SS and NASH model mice

A total of 18 male C57/BL6 mice, aged 6-8 weeks, were randomly assigned to three groups. The first group received a high-fat diet (HFD) consisting of 60% fat (n = 6), the second group was fed on an MCD diet (n = 6), and the third group was given a control diet (CD) with 10% fat (n = 6) for a duration of 8 weeks. At the Second Affiliated Hospital of Harbin Medical University, the animals were kept in a controlled environment where the temperature was regulated. They were provided with food and water freely and followed a 12-hour cycle of light and darkness. The animal experiments received ethical clearance from the Ethics Committee of Second Affiliated Hospital of Harbin Medical University (SYDW2023-077).

### 2.10 Histological analysis

Liver tissues were isolated from mice and immediately fixed with 4% formalin (Sigma- Aldrich, St. Louis, MO). Afterward, the dehydrated samples were embedded in paraffin. Histological changes were examined by H&E staining. Images were acquired using the Eclipse E100 microscope (Nikon, Japan). Mice models were evaluated by two qualified pathologists.

### 2.11 Quantitative RT-PCR analysis

Total RNA was extracted from liver tissues using Trizol reagent (Invitrogen), and then reverse transcribed into cDNA using the Transcriptor First Strand cDNA Synthesis Kit (Roche, Penzberg, Germany). FastStart Universal SYBR Green Master (Roche) was used to amplify each sample in a reaction mixture of 20 μl. The fold changes were converted using the 2-ΔΔCt technique. Expression levels were determined by calculating and normalizing them to the endogenous GAPDH. The primer sequences were shown in **Table 1**.

**Table 1:** The sequences of primers.

Genes	Forward Primer (5′-3′)	Reverse Primer (5′-3′)
GAPDH	GTGCCGCCTGGAGAAAC	AAGGTGGAAGAGTGGGAGT
Jun	GGGAGCATTTGGAGAGTCCC	TTTGCAAAAGTTCGCTCCCG
Ccl20	CCAGGCAGAAGCAAGCAAC	TTTGGATCAGCGCACACAGA

### 2.12 Statistical analysis

The findings were presented as average ± standard deviation from a minimum of 3 separate trials. The differences between groups were compared using t-test, and GraphPad Prism 8.0 and R software version 4.2.3 were utilized for data analyses. Statistical significance was determined by comparing p-values, where p < 0.05 denoted significance (*p < 0.05, **p < 0.01, ***p<0.001, ****p<0.0001).

## 3 Result

### 3.1 Weighted gene co-expression network construction

**Figure 2:**
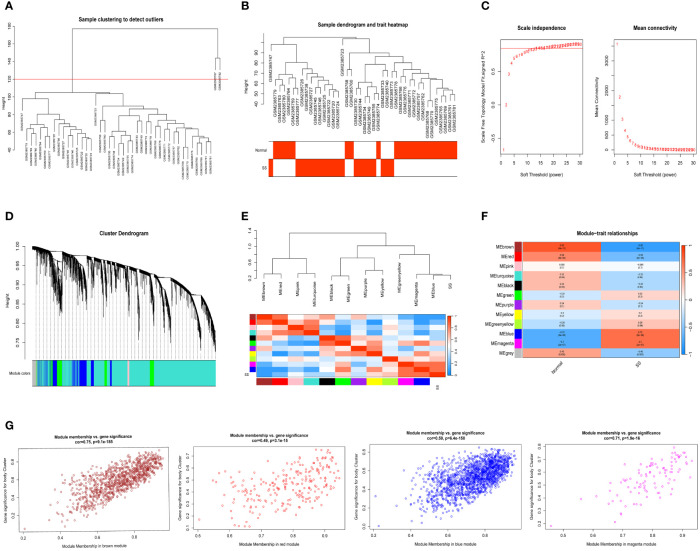
Detection of module genes using WGCNA in normal vs. SS group. **(A)** Removal of outlier samples from normal vs. SS group. **(B)** The clustering was performed using the expression data of the normal vs. SS groups, with the color intensity indicating the disease status (normal and SS). **(C)** The “soft” threshold was chosen based on the combined analysis of scale independence and average connectivity. **(D)** Different colors represent gene coexpression modules in the gene tree. **(E)** Collinear heat map of module feature genes. A high correlation is indicated by the color red, while opposite results are indicated by the color blue. **(F)** A graphical representation showing the relationship between modules and traits, with each cell displaying the correlation and P value associated with it. **(G)** Scatter plot illustrating the relationship between MM and GS in the top four modules.

We selected data of normal and SS patients in the dataset to identify regulatory genes related to the occurrence of SS. Correlation networks were used for identifying clusters of highly correlated genes across microarray samples. We employed WGCNA to construct and analyzed active SS-associated networks. After clustering the samples, we established a suitable threshold (cutHeight = 120) to eliminate the clearly abnormal samples (**Figure 2A**). After removing outliers, we drew a sample clustering tree (**Figure 2B**). Here, we selected top 10000 genes for subsequent analysis. Using a soft-threshold power of β = 14 (R2 = 0.85), the adjacency matrix was created, ensuring gene distribution adhered to a scale-free network (**Figure 2C**). This retained valuable connectivity information. A total of 11 modules were generated and identified under the parameter settings of minModuleSize = 30 and mergeCutHeight = 0.25 (**Figure 2D**). The connectivity was calculated among the modules and we added group information to them, then we performed the cluster analysis, meanwhile, the heat map of their correlation was also plotted (**Figure 2E**). In order to further examine the connection between the models and phenotype, we computed the correlation coefficients of each model with the SS trait. The findings indicated that SS had a statistically significant correlation with 6 modules. Among them, SS is highly associated with four modules: ‘brown’ (r = -0.82, p = 4e−11), ‘red’ (r = -0.58, p = 6e−05), ‘blue’ (r = 0.72, p = 8e−08), and ‘magenta’ (r = 0.7, p = 2e−07) (**Figure 2F**). Next, we conducted an analysis of module membership (MM) and gene significance (GS) correlation for these 4 modules. Interestingly, we found a strong positive correlation between MM and GS in these 4 modules (**Figure 2G**). After combining the genes of these four modules, we ultimately acquired a sum of 2939 genes within the modules.

### 3.2 Identification of immune related key genes involved in the onset and progression of NAFLD

**Figure 3:**
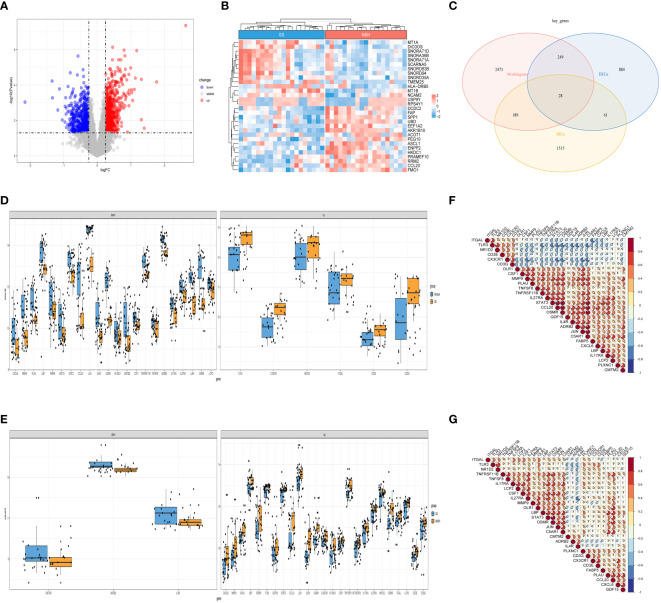
Detection of DEGs in the SS vs. NASH group and identification of immune related key genes. **(A)** The volcano plot of DEGs in SS vs. NASH group. **(B)** Heat map displayed the top 30 genes that show significant differences. **(C)** The immune related key genes were acquired by intersecting the module genes obtained through WGCNA in the normal vs. SS group, the DEGs from the SS vs. NASH group, and the IRGs. **(D, E)** The variations in the manifestation of 28 immune related key genes between the normal and SS group, as well as the SS and NASH group. **(F, G)** The correlation between 28 immune related key genes in normal vs. SS group and SS vs. NASH group.

Next, we selected data of SS and NASH patients in the dataset to identify regulatory genes related to the development of SS. DEGs were identified by converting the fold changes (FC) of gene expression to log2 values and applying the cutoff criteria of |log2FC| ≥ 0.25 and p-value < 0.05. Based on these criteria, a total of 1222 genes were identified as DEGs that potentially contribute to the progression of SS. Among these genes, 694 were found to be up-regulated while 528 were down-regulated. Volcano plots of DEGs were displayed in **Figure 3A**, while **Figure 3B** presented a heat map showcasing the top 30 genes. By intersecting the DEGs, module genes, and IRGs, we identified a total of 28 genes that were present in all three gene clusters during the onset and progression of NAFLD (**Figure 3C**). Box plots displayed the variations in gene expression between the normal vs. SS group, as well as the SS vs. NASH group. We could observe that 6 genes were upregulated in SS patients, while 22 genes were upregulated in normal samples (**Figure 3D**). By contrast, most of these 28 genes were highly expressed in NASH patients compared to patients with SS (**Figure 3E**). In addition, we also analyzed the correlation between these genes, and the results were shown in **Figures 3F, G**, which suggested that most of the genes might interact with each other and participate in the same pathway.

### 3.3 Enrichment analyses of 28 immune related key genes

**Figure 4:**
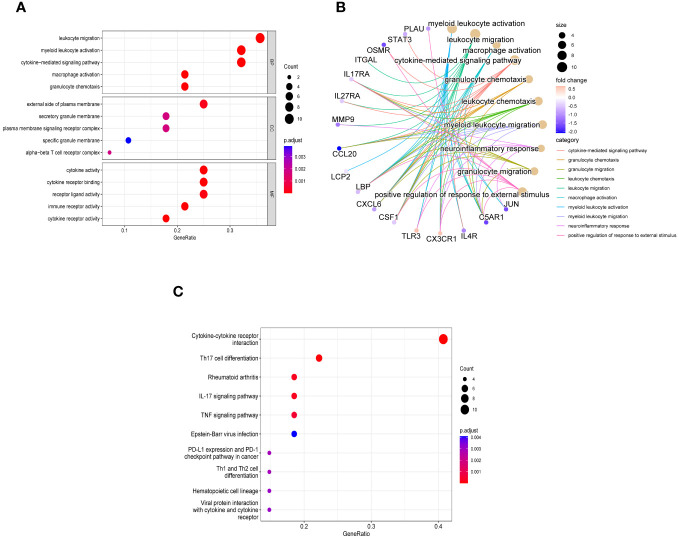
Enriched items in GO and KEGG analyses of 28 immune related key genes. **(A)** The enriched terms in GO analysis. **(B)** Correlation between the top 10 biological functions and genes. **(C)** KEGG analysis.

In order to explore the biological functions and pathways of these 28 immune related key genes, GO and KEGG enrichment analyses were performed. We obtained a total of 298 biological processes that were significantly related, along with 23 KEGG signaling pathways. To uncover the biological functions of immune-related key genes, a GO analysis was conducted (**Figure 4A**). As observed, the majority of genes in the GO category were primarily involved in functions such as ‘leukocyte migration’, ‘myeloid leukocyte activation’, ‘cytokine-mediated signaling pathway’, ‘macrophage activation’, and ‘granulocyte chemotaxis’ (BP); ‘external side of plasma membrane’, ‘secretory granule membrane’, ‘plasma membrane signaling receptor complex’, ‘specific granule membrane’, and ‘alpha-beta T cell receptor complex’ (CC); ‘cytokine activity’, ‘cytokine receptor binding’, ‘receptor ligand activity’, ‘immune receptor activity’, and ‘cytokine receptor activity’ (MF). **Figure 4B** showed the correlation between the top 10 biological functions and genes. According to the KEGG pathway enrichment analysis, the Cytokine-cytokine receptor interaction, Th17 cell differentiation, Rheumatoid arthritis, IL-17 signaling pathway, TNF signaling pathway, and other pathways were found to be highly associated with immune response and inflammation (**Figure 4C**).

### 3.4 Analysis of the network of interactions between proteins

**Figure 5:**
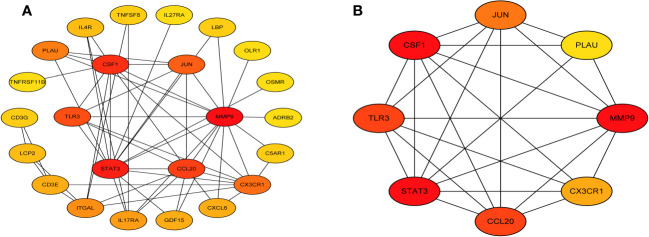
Visual representation of the protein-protein interaction networks. **(A)** PPI network of immune related key genes. **(B)** Gene clustering based on the MCODE algorithm.

Next, we accessed the STRING database and constructed a PPI network for the immune related key genes using Cytoscape software, 4 genes were kicked out because they had no interaction with the other genes, including CMTM2, PLXNC1, NR1D2, and FABP5 (**Figure 5A**). MCODE was used to explore the most significant cluster (cluster 1, containing 8 genes). Using the MCC algorithm, the interaction network (included 8 nodes and 48 edges) of these 8 genes were obtained through the CytoHubba plugin of the software Cytoscape (**Figure 5B**).

### 3.5 Integrated LASSO analysis, RF algorithm, and SVM for screening hub genes

**Figure 6:**
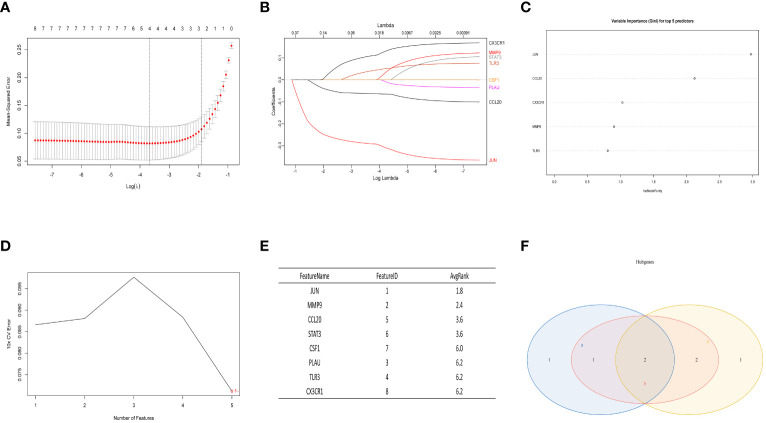
The LASSO analysis, RF algorithm, and SVM were used to identify the ultimate hub genes. **(A, B)** LASSO regression analysis. **(C)** RF algorithm. **(D, E)** Machine learning approach with SVM. **(F)** Venn diagram showing the central genes identified by LASSO, SVM-RFE, and RF.

In the normal vs. SS group, the LASSO Cox regression model was employed to identify the most valuable diagnostic gene signature among the mentioned genes, resulting in the identification of 4 potential genes (**Figures 6A, B**). Next, the RF algorithm evaluated the significance of each gene and determined the ranking of these 8 genes. From this ranking, we selected the top 5 genes with the highest importance (as shown in **Figure 6C**). Simultaneously, we utilized a machine learning technique with SVM to conduct a thorough analysis of the distinct genes, and the findings indicated that opting for the top five genes is suitable (**Figures 6D, E**). Finally, we intersected the three screening results mentioned above and obtained the optimal gene signature consisting of 2 diagnostic genes, including JUN and CCL20 (**Figure 6F**).

### 3.6 Expression and diagnostic efficacy identification of hub genes

**Figure 7:**
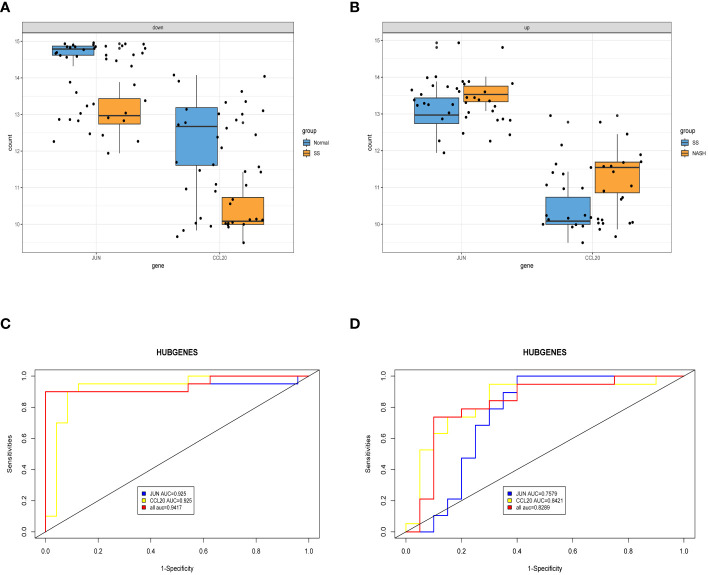
Exploring the expression levels and predictive value of hub genes. **(A, B)** Expression levels of two hub genes in normal vs. SS group and SS vs. NASH group, respectively. **(C, D)** ROC analysis of two hub genes in normal vs. SS group and SS vs. NASH group, respectively.

After conducting an examination, we observed the expression of two central genes derived from the aforementioned analysis in the normal vs. SS group as well as the SS vs. NASH group. Surprisingly, their expressions were entirely contrasting, indicating that these two genes potentially perform contradictory functions during various phases of the ailment (**Figures 7A, B**). In order to confirm the diagnostic significance of the two central genes, we generated ROC curves and determined the area under the curve (AUC) for these genes. In normal vs. SS group, the AUC of both two genes were 0.925, while the combined diagnostic efficacy of the two genes was better than that of any single one (AUC: 0.9417) (**Figure 7C**). In SS vs. NASH group, the AUC of JUN and CCL20 were 0.7579 and 0.8421, respectively, however, the combined diagnostic efficacy of the two genes was not improved (AUC: 0.8289) (**Figure 7D**).

### 3.7 Validation of hub genes expression and diagnostic efficacy through a GEO dataset and mouse model

**Figure 8:**
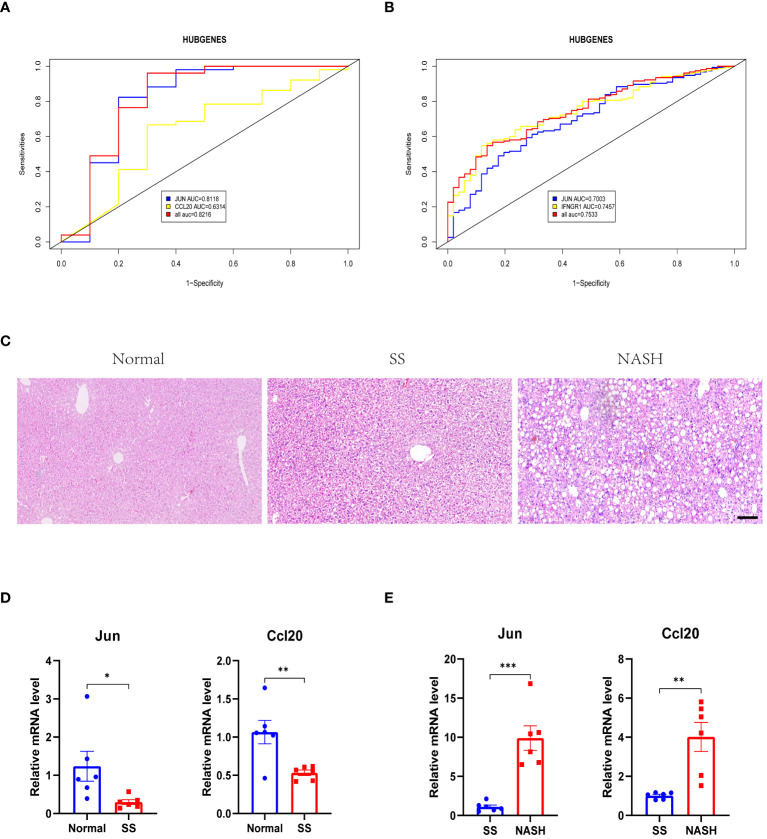
Verification of the two hub genes. **(A, B)** ROC analysis of two hub genes through dataset GSE135251. **(C)** H&E staining of liver slides. **(D, E)** The expression patterns of two hub genes through mouse model. Scale bar, 100 um. *p < 0.05; **p < 0.01; ***p < 0.001.

For the purpose of confirming the expression patterns and diagnostic effectiveness of hub genes, the dataset GSE135251 was utilized in the current investigation. The findings indicated that the expression of hub genes aligned with the aforementioned outcomes (**Table 2**). In addition, in the dataset GSE135251, both genes had good diagnostic efficacy (**Figures 8A, B**). During the animal study, H&E staining showed obvious fat accumulation in the SS and NASH groups, and a large number of immune cell infiltration in the NASH group (**Figure 8C**). In comparison to the normal group, we observed a significant decrease in the expression of the two hub genes in the SS group. On the contrary, compared with SS group, the expression levels of these two genes were significantly up-regulated in NASH group. **Figures 8D, E** displayed the relative mRNA expression of the two genes.

**Table 2:** The gene expression pattern in dataset GSE135251 for hub genes.

Normal vs. SS			SS vs. NASH		
Gene Symbol	logFC	Adjusted p-Value	Gene Symbol	logFC	Adjusted p-Value
JUN	-1.4096286	6.73e-05	JUN	0.4997750	9.93e-04
CCL20	-0.1656848	2.63e-01	CCL20	0.5086908	2.36e-04

### 3.8 Analysis of immune infiltration and correlation analysis

**Figure 9:**
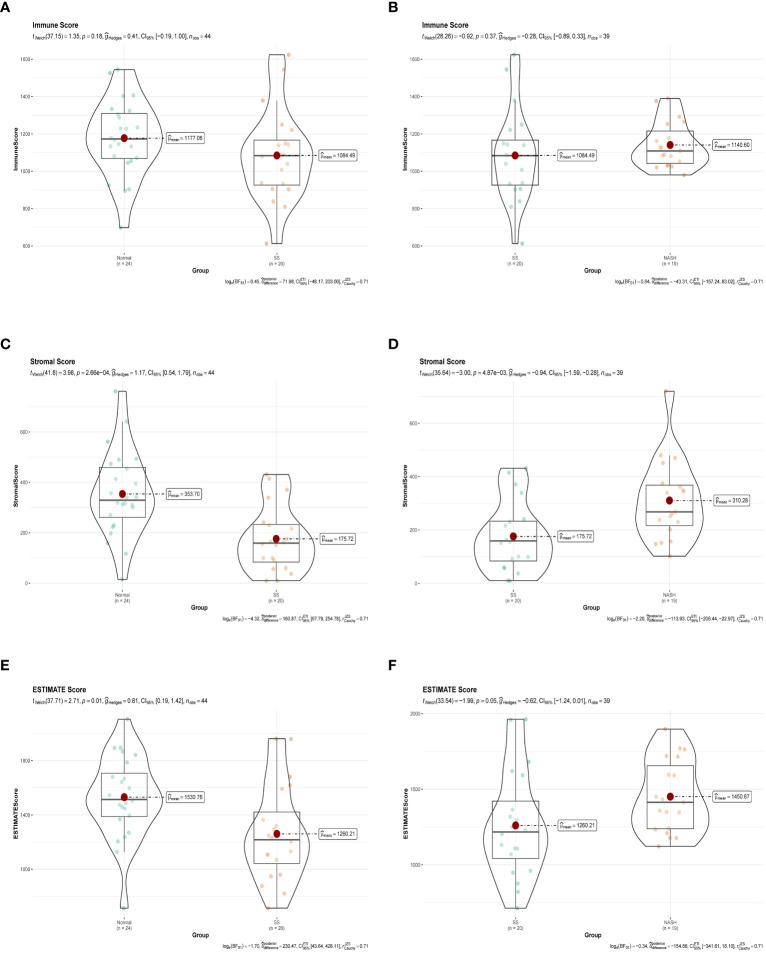
Immune status assessment by ESTIMATE algorithm. **(A, B)** Immune Score in normal vs. SS group and SS vs. NASH group, respectively. **(C, D)** Stromal Score in normal vs. SS group and SS vs. NASH group, respectively. **(E, F)** ESTIMATE Score in normal vs. SS group and SS vs. NASH group, respectively.

**Figure 10:**
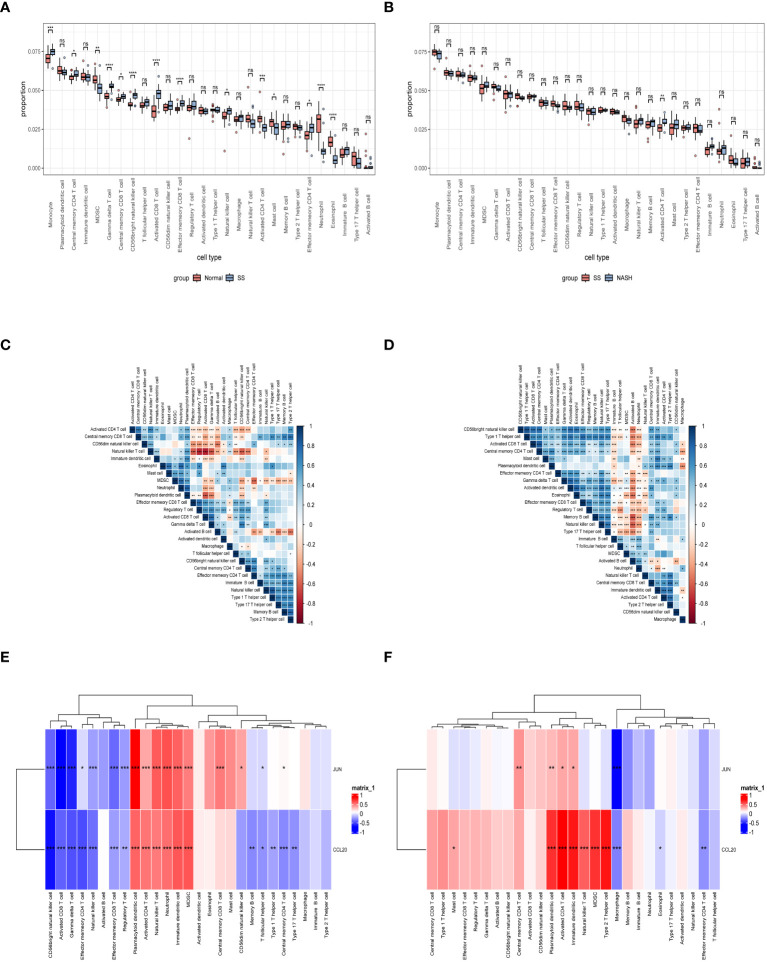
Exploration of immune infiltration in NAFLD and its association with central genes through ssGSEA. **(A, B)** Analysis of the immunocyte infiltration degrees regarding 28 immunocyte subunits in normal vs. SS group and SS vs. NASH group, respectively. **(C, D)** Correlation between different immune cells in SS and NASH patients, respectively. The correlation between immune cell infiltration and two hub genes in patients with SS and NASH, respectively. *p < 0.05; **p < 0.01; ***p < 0.001; ns: no significance.

Immune dysregulation in the liver microenvironment could potentially be linked to the onset and progression of NAFLD. Hence, in order to comprehend the development of SS and its progression to NASH, the ESTIMATE algorithm was utilized to deduce the infiltration of immune cells. **Figures 9A, C, E** demonstrated that individuals with SS exhibited decreased immune score, stromal score, and ESTIMATE score in comparison to the healthy controls. In comparison, NASH patients exhibited elevated immune score, stromal score, and ESTIMATE score in contrast to SS patients (**Figures 9B, D, F**). Furthermore, we employed ssGSEA to assess the variances in the abundance of 28 immune cell subpopulations infiltrating the hepatic tissue between the normal and SS groups, as well as the SS and NASH groups, using data from the GSE89632 dataset (**Figures 10A, B**). Compared to healthy controls, SS patients exhibited higher levels of immune cell infiltration, including monocytes, central memory CD4 T cells, gamma delta T cells, central memory CD8 T cells, CD56 bright natural killer cells, activated CD8 T cells, effector memory CD8 T cells, natural killer cells, and effector memory CD4 T cells, in the normal vs. SS group. On the contrary, MDSCs, activated CD4 T cells, mast cells, neutrophils, and eosinophils were enriched in healthy controls. In SS vs. NASH group, high infiltration level of activated CD4 T cells was observed in NASH patients compared to SS patients. Based on **Figures 10C, D**, we conducted a correlation analysis on immune cells in both SS and NASH patients, revealing noteworthy associations among the majority of immune cells in these individuals. We conducted separate correlation analyses to investigate the connection between our identified hub genes JUN and CCL20 and the content of immune cells. Among individuals with SS, there was a robust positive association between JUN expression and Plasmacytoid dendritic cell (r = 0.890), whereas the CCL20 expression exhibited a noteworthy inverse relationship with CD56 bright natural killer cell (r = -0.686) (**Figure 10E**). Among individuals with NASH, there was a notable inverse relationship between JUN expression and macrophage, with a correlation coefficient of -0.611. Conversely, the CCL20 expression exhibited a robust positive correlation with activated CD4 T cell, with a correlation coefficient of 0.767 (as shown in **Figure 10F**). Collectively, this suggested that these central genes facilitated the immune response throughout the progression of the disease in individuals with NAFLD.

## 4 Discussion

NAFLD is a complex disease caused by multiple factors, especially obesity. Previous studies have extensively explored its pathogenesis and found that the mechanism of its development was affected by a variety of factors, such as age, menopause, and type 2 diabetes (T2D) (25, 26). Since the accumulation of fat may affect the infiltration of immune cells, the abnormal function of the immune system in NAFLD has been paid more and more attention (27, 28). Song et al. found that C/EBPα was significantly upregulated in NASH compared with healthy controls, possibly contributing to disease progression by regulating intrahepatic immune and inflammatory responses (29). In addition, multiple research studies validate that both inherent and acquired immune disorders are significant contributors to the development and advancement of NAFLD, for example, during NASH, there is a large infiltration of neutrophils and NKT cells in the liver, which is closely related to the development of the disease in the direction of increased inflammation (30–32). Moreover, platelets in the liver interact with Kupffer cells to induce the secretion of alpha granules, which contains large amounts of growth factors as well as cytokines, thereby exacerbating liver inflammation (33). Modifying the expression of IRGs brings about variations in the infiltration level and functional state of immune cells (34). Disorders of IRGs have been described in a variety of diseases, such as osteosarcoma and Alzheimer’s disease (35, 36). Considering these factors, we implemented a thorough and extensive assessment system to examine the immune-associated hub genes and molecular pathways in the onset and progression of NAFLD using bioinformatics. Our objective is to expand the understanding of the physiological pathology and molecular mechanisms of NAFLD, and offer insights for clinical diagnosis and treatment approaches.

For this particular investigation, we obtained the gene expression matrix of SS tissue in comparison to normal liver tissue and NASH tissue in comparison to SS tissue from the GSE89632 dataset. In normal vs. SS group, we obtained 11 SS related modules using WGCNA, and after further screening, we identified 4 modules that were strongly correlated with SS, and finally obtained 2939 module genes (**Figure 2**). In SS vs. NASH group, we performed differential analysis using another analytical method and obtained 1222 DEGs, of which 694 genes were up-regulated and 528 genes were down-regulated. By intersecting module genes, DEGs, and IRGs, we identified 28 immune-related genes that played a role in both the onset and progression of NAFLD (**Figure 3C**). Previous studies performed high-throughput sequencing of liver tissue from normal mice and mice at different stages of NASH, they found that compared with normal liver tissue, the expression patterns of genes in the liver tissue of model mice and the signaling pathways involved changed as the disease progressed (37). In our study, we performed a follow-up analysis to determine whether the expression patterns of these 28 genes also changed as the NAFLD progressed. Additional examination of the comparative expression of these 28 genes uncovered a fascinating observation that these genes did not consistently exhibit increased or decreased regulation at the initiation of NAFLD and the advancement to NASH (**Figures 3D, F**). This suggested that the immune microenvironment might be diametrically opposed at different stages of the disease. Analysis of the immune infiltration score further confirmed our conjecture (**Figure 9**). Dynamic changes in immune cell infiltration in the immune microenvironment at different stages of the disease had previously been demonstrated in a variety of diseases (38, 39). In addition, this also suggested that the treatment might not be consistent at different stages of the disease.

In order to obtain a deeper understanding of the possible roles of these genes associated with the immune system, we utilized bioinformatics tools to conduct GO and KEGG enrichment analyses on the set of 28 genes. Analysis of the genes revealed their involvement in immune-related pathways, particularly cytokine-related pathways and the activation and chemotaxis of immune cells (**Figure 4**). This could explain the lower infiltration of activated CD4 T cells during the early stage of the disease and their higher level in NASH. And through a variety of bioinformatics analysis methods, such as PPI, LASSO, RF, and SVM, we further screened from these 28 genes and obtained 2 hub genes, including JUN and CCL20.

JUN plays a vital role in activator protein 1 (AP-1), being essential for liver development and contributing to the onset and progression of diverse liver disorders (40). Previous studies focused on the differential expression of this molecule between NAFLD and normal healthy controls, but the changes in the expression level of this molecule at different stages of the disease and its remodeling effect on the liver immune microenvironment were rarely mentioned (41–43). During this investigation, it was discovered that JUN exhibited a decrease in expression levels in SS in comparison to individuals without any health issues. This was consistent with the findings of Qu et al., however, they did not further explore the expression pattern of JUN in NASH stage (44). Our study had found that as the disease advanced to NASH, JUN was notably up-regulated. Furthermore, this particular molecule played a crucial role in governing the infiltration of various immune cells throughout different phases of the disease.

CCR6^+^ cells are driven to migrate through tissues by the high affinity binding of CCL20 to its receptor CCR6, which is currently the sole known ligand for CCR6 (45). Increased infiltration of CCR6^+^ lymphocytes with upregulated CCL20 expression was found in tissue microenvironment during inflammation, infection and malignant lesions in various organs, such as stomach, intestine, liver and lung (46). In addition, one study found that postmenopausal women with T2D were more likely to have upregulated CCL20 expression levels, which might be closely related to a more pronounced liver inflammatory response and susceptibility to NASH in these patients (25). In this study, we found that CCL20 was down-regulated in SS compared with healthy controls, but significantly up-regulated when the disease progressed to NASH. In our research, it was discovered that this particular molecule exhibited a strong positive correlation with the infiltration of activated CD4 T cells in NAFLD. Earlier research had validated that stimulated CD4 T cell types, like Th1 and Th17, possess the ability to release diverse cytokines, including IFN-γand IL-17, thereby enhancing liver inflammation (47–49). Hence, we hypothesized that this compound might have a crucial function in the progression of SS to NASH.

HFD-induced mice and MCD-induced mice are currently recognized animal models that can mimic human NAFLD, and these models are highly similar to human SS and NASH in histologic appearance and liver transcriptome characteristics (50, 51). Therefore, in our study we constructed the above two models. Afterwards, we confirmed the gene expression by conducting *in vivo* experiments, the results aligned with the sequencing findings. Simultaneously, we also confirmed the expression of these two compounds by analyzing an additional dataset, GSE135251, and the outcome aligned with our discoveries. We then further explored the diagnostic capabilities of JUN and CCL20 and found that they were able to distinguish between different stages of NAFLD disease. In summary, JUN and CCL20 had been identified as key targets for mediating the onset of NAFLD and leading to its progression from SS to NASH, and may be markers for predicting disease progression.

In conclusion, we proposed that there were dynamic changes in the expression levels of IRGs in different stages of NAFLD disease, and they regulated the immune microenvironment of the liver mainly through the cytokine related pathways and immune cell activation and chemotaxis, which suggested that the treatment might not be consistent at different stages of the disease. Among them, JUN and CCL20 might be the key molecules that promoted the occurrence and progression of the disease, and had potential as diagnostic markers and therapeutic targets. However, our study was based on the public database, therefore, further experiments were needed to explore and verify the mechanism of the results obtained from the analysis. We believe these will further deepen our understanding of the disease and provide references for the treatment of the disease.

## Data availability statement

The original contributions presented in the study are included in the article/supplementary material. Further inquiries can be directed to the corresponding authors.

## Ethics statement

The animal study was approved by the Ethics Committee of Second Affiliated Hospital of Harbin Medical University. The study was conducted in accordance with the local legislation and institutional requirements.

## Author contributions

RH: Writing – original draft, Data curation. CG: Writing – original draft, Data curation. XZ: Writing – review & editing. LY: Writing – review & editing. YC: Writing – review & editing.
